# Supplementary material for: Novel Ubiquitin Specific Protease-13 Inhibitors Alleviate Neurodegenerative Pathology
Source: Metabolites. 2021 Sep 15;11(9):622. doi: 10.3390/metabo11090622 (PMC8467576; doi:10.3390/metabo11090622)
Supplement: Supplementary file 1 [file metabolites-11-00622-s001.zip › metabolites-1337266-supplementary.pdf]

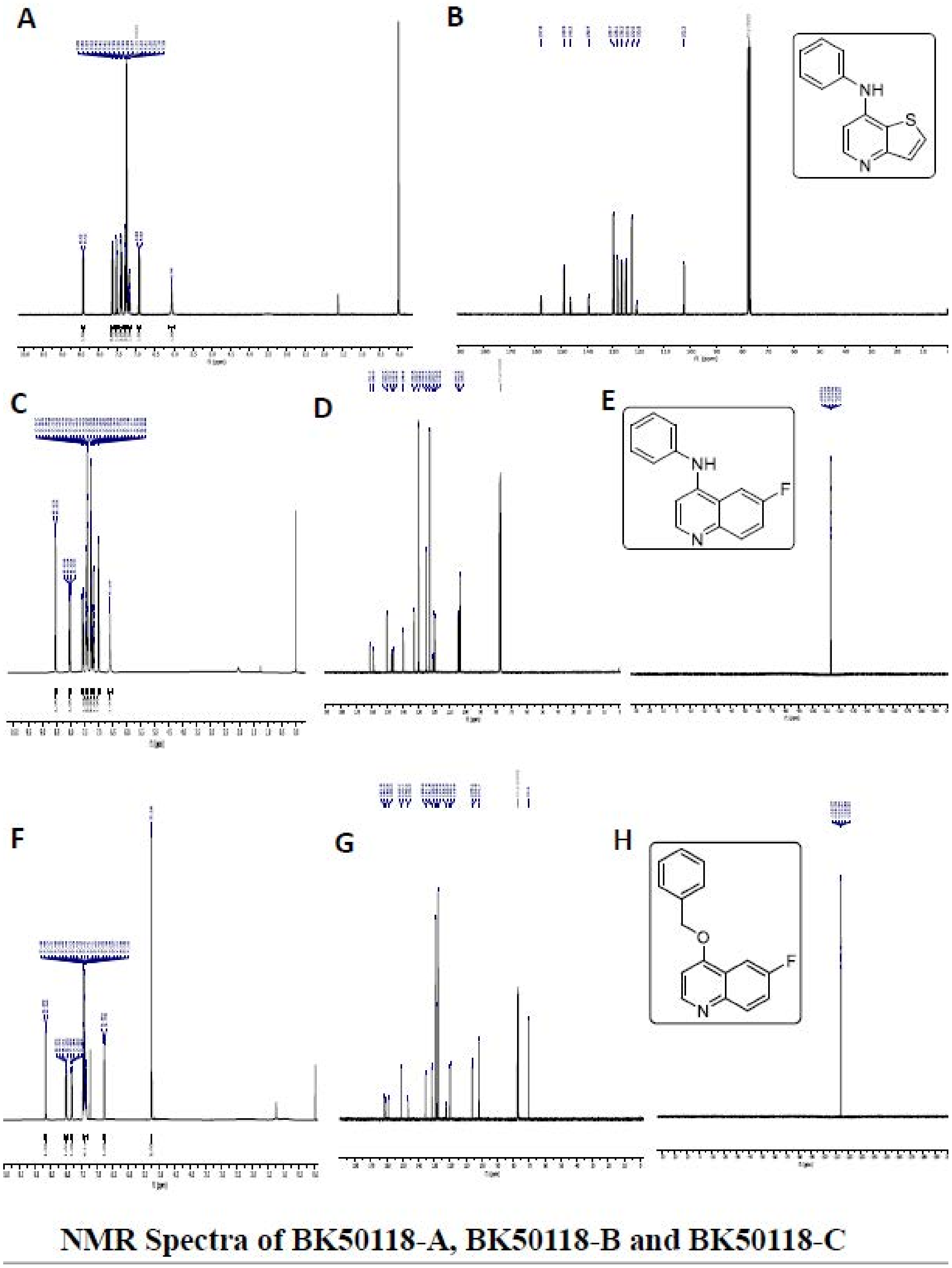

Suppl Figure S1. NMR Spectra of BK50118-A, BK50118-B and BK50118-C.

A 1H NMR Spectrum of N-phenylthieno[3,2-b]pyridin-7-amine (BK50118-A).

B 13C NMR Spectrum of N-phenylthieno[3,2-b]pyridin-7-amine (BK50118-A).

C 1H NMR Spectrum of 6-fluoro-N-phenylquinolin-4-amine (BK50118-B).

D 13C NMR Spectrum of 6-fluoro-N-phenylquinolin-4-amine (BK50118-B).

E 19F NMR Spectrum of 6-fluoro-N-phenylquinolin-4-amine (BK50118-B).

F 1H NMR Spectrum of 4-(benzyloxy)-6-fluoroquinoline (BK50118-C).

G 13C NMR Spectrum of 4-(benzyloxy)-6-fluoroquinoline (BK50118-C).

H 19F NMR Spectrum of 4-(benzyloxy)-6-fluoroquinoline (BK50118-C).

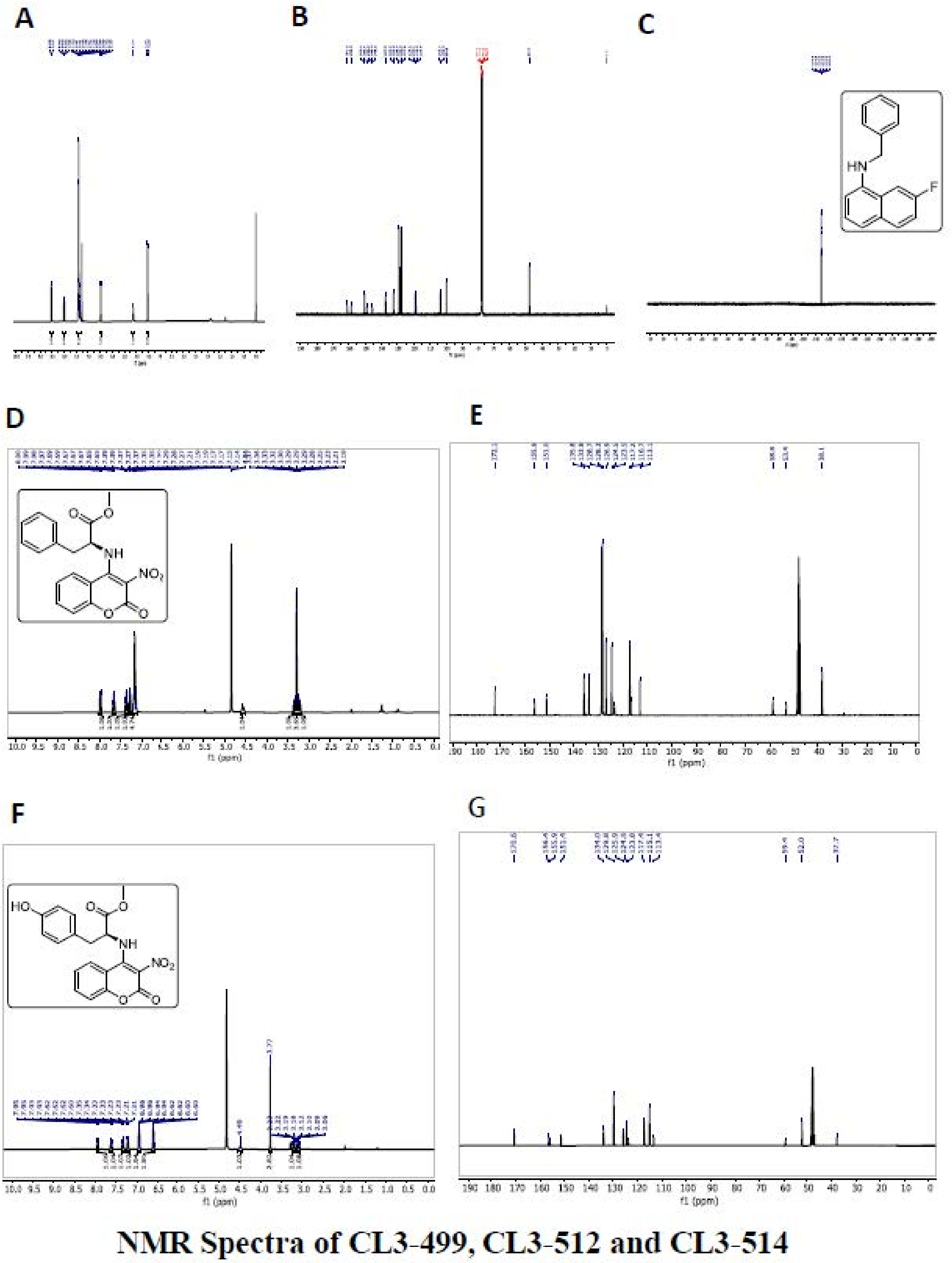

Suppl Figure S2. NMR Spectra of CL3-499, CL3-512 and CL3-514.

A 1H NMR Spectrum of N-benzyl-6-fluoroquinolin-4-amine (CL3-499).

B 13C NMR Spectrum of N-benzyl-6-fluoroquinolin-4-amine (CL3-499).

C 19F NMR Spectrum of N-benzyl-6-fluoroquinolin-4-amine (CL3-499).

D 1H NMR Spectrum of (S)-methyl (3-nitro-2-oxo-2H-chromen-4-yl)-phenylalaninate (CL3-512).

E 13C NMR Spectrum of (S)-methyl (3-nitro-2-oxo-2H-chromen-4-yl)-phenylalaninate (CL3-512).

F 1H NMR Spectrum of (S)-methyl (3-nitro-2-oxo-2H-chromen-4-yl)-tyrosinate (CL3-514).

G 13C NMR Spectrum of (S)-Methyl (3-nitro-2-oxo-2H-chromen-4-yl)-tyrosinate (CL3-514).

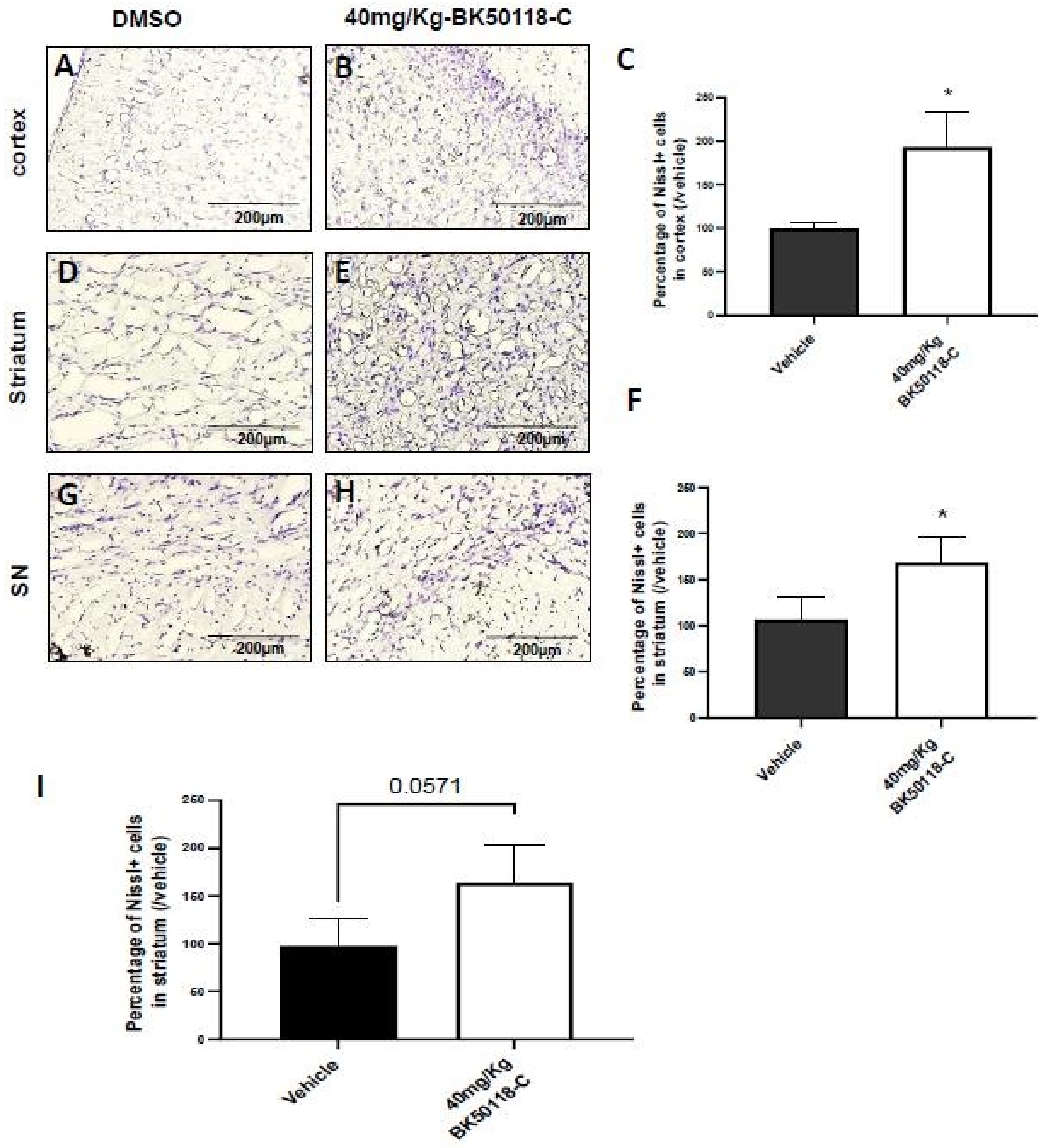

Suppl Figure S3. BK50118-C improved cell survivals in TgA53T mice. Male and female TgA53T mice were treated with intraperitoneal injection of vehicle or BK50118-C at the daily dosage of 40 mg/Kg for 7 days. Nissl staining showed BK50118-C significantly increased the neuron counts in cortex **B)**, striatum **E)** and substantia nigra (SN) **H)** compared to corresponding vehicles **A), D) and G),** verified

by quantification of Nissl+ cells in cortex **C),** striatum **F)** and SN **I)**. Asterisk indicates statistic significant difference vs vehicle. * $p<0.05$ vs vehicle. Two-tailed student's t test was used for analysis. N=3-4 mice per group. All values presented as Mean±SD. Scale bars: 200μm.

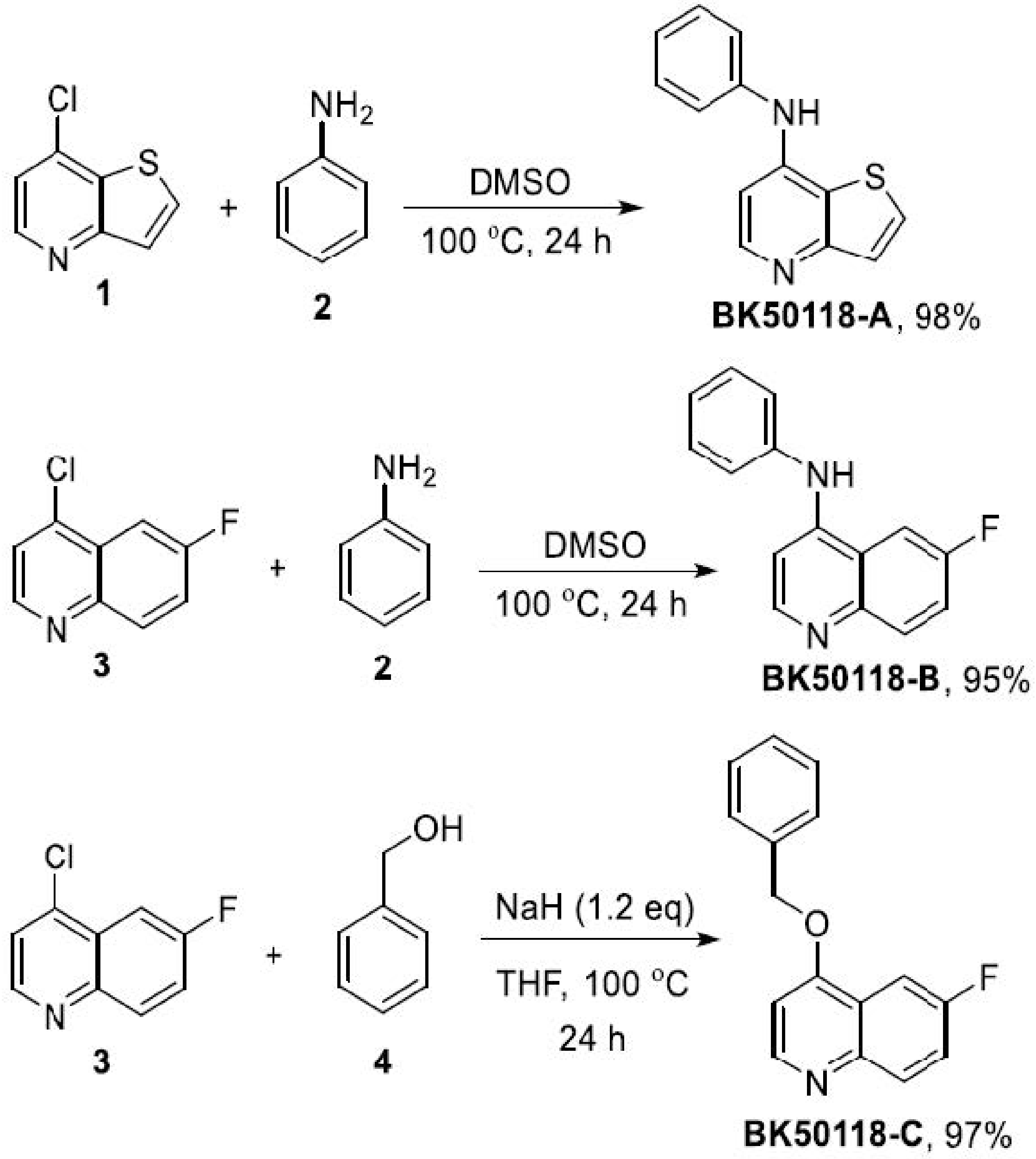

Synthesis of BK50118-A, BK50118-B and BK50118-C

Suppl Figure S4. Synthesis of BK50118-A, BK50118-B and BK50118-C.

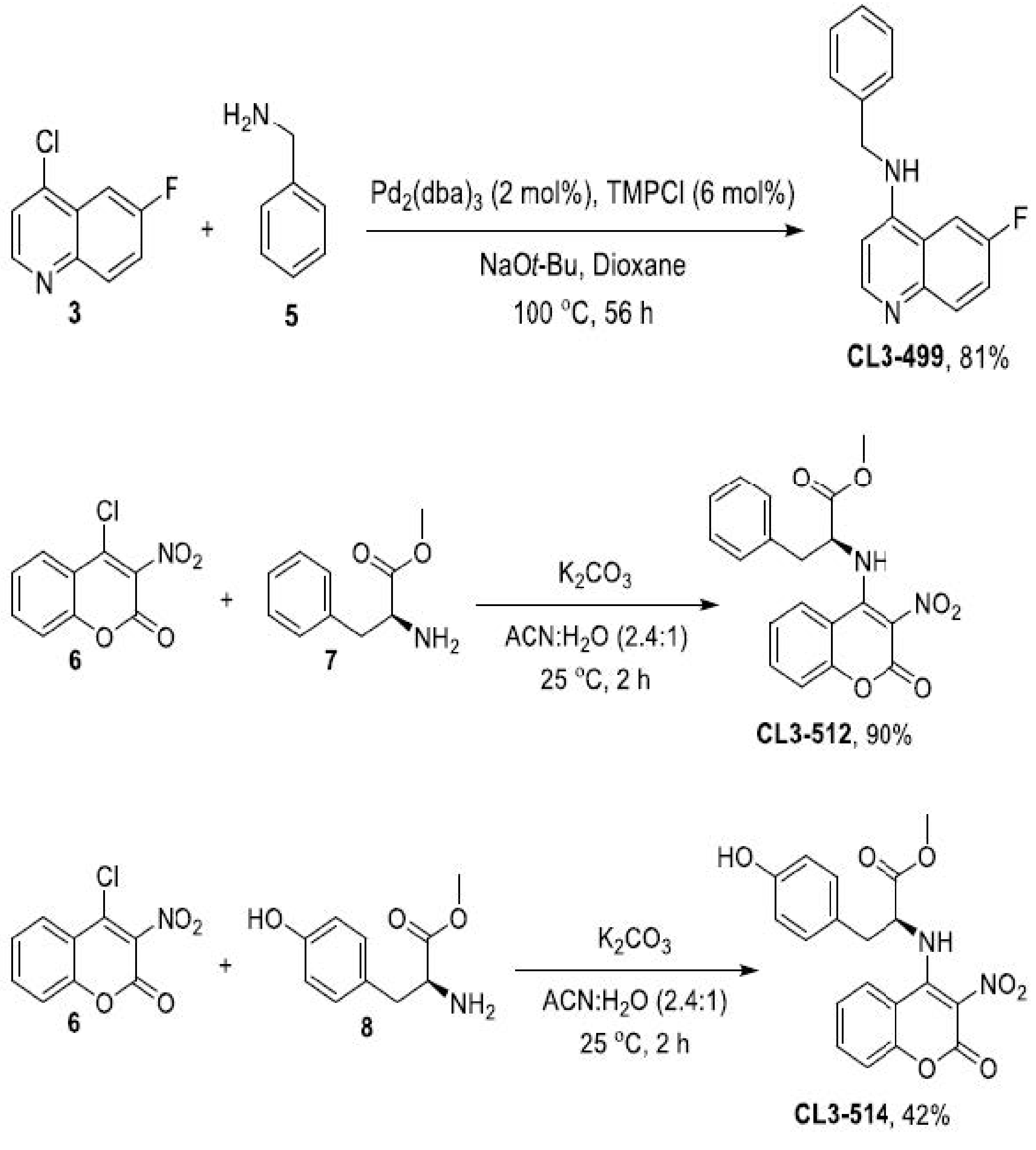

Synthesis of CL3-499, CL3-512 and CL3-514

Suppl Figure S5. Synthesis of CL3-499, CL3-512 and CL3-514.
